# When two signals are better than one: Synergistic control of erythropoiesis

**DOI:** 10.1371/journal.pbio.3003512

**Published:** 2025-12-12

**Authors:** Nicola K. Wilson

**Affiliations:** Department of Haematology, Cambridge Stem Cell Institute, University of Cambridge, Cambridge, United Kingdom

## Abstract

Red blood cell production is one of the most dynamic processes, yet the underlying mechanisms responsible are only partially understood. A new study in PLOS Biology suggests a broadly applicable mechanism able to balance the maintenance of the steady-state and effective stress response.

Red blood cells constitute over 80% of the total cells of the body; they are responsible for the transport of oxygen from the lungs to every tissue of the body and undergo a high rate of cellular turnover. Erythropoiesis is extremely dynamic and requires high levels of resources from the organism. Anemia is a common occurrence if the organism experiences disease, inflammation, or nutritional deficiencies. Therapeutic options for the treatment of anemia are limited due to the lack of insight into the factors which can stimulate erythropoiesis. In this issue of PLOS Biology, Wu and colleagues [1] present insights into a broadly applicable mechanism by which pleiotropic cytokines achieve lineage-specific effects through synergistic interactions with lineage-specific factors.

The stepwise process of red blood cell production is well understood, in that hematopoietic stem and multipotent progenitors differentiate into intermediate amplifying progenitors, which then progress towards erythroid terminal differentiation [2], a process which is tightly regulated by erythropoietin. Additional factors such as stem cell factor and BMP4 [3,4], along with glucocorticoid receptor [5], have been shown to modulate stress erythropoiesis, defined as the enhanced production of erythroid cells in response to anemic stress. However, more detailed knowledge and better understanding of the downstream mechanisms will be important to understand the regulation of erythropoiesis and understand how these mechanisms can be manipulated in targeted therapeutics to improve erythropoiesis in stress conditions. A key additional consideration is how to modulate ineffective erythropoiesis—characterized by the expansion of early erythroid precursors in the bone marrow coupled with impaired maturation and apoptosis of late-stage erythroid precursors—without adversely affecting other hematopoietic and immune lineages within the organism.

Following on from previous work, which identified the expression of additional growth factors and cytokine receptors on the surface of erythroid progenitors [6], Wu and colleagues [1] investigated the role of the pro-inflammatory cytokine IL-17A. Using a detailed phenotypic analysis of bone marrow and spleen, the authors clearly demonstrate that administration of IL-17A alone was unable to promote erythropoiesis, yet a striking synergistic interaction was observed when administered in combination with erythropoietin (Epo). To investigate the mechanism underpinning the synergistic action of Epo and IL-17A, the authors performed transcriptomic analysis, which unexpectedly did not highlight a unique gene signature following IL-17A exposure, but rather provided evidence that IL-17A exposure was able to amplify Epo-dependent gene expression, thereby sensitizing early erythroid progenitors to Epo (Fig 1).

**Fig 1:**
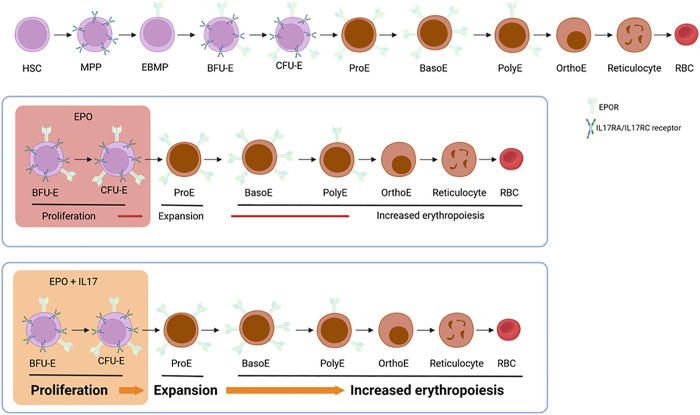
Synergistic mechanisms of pleiotropic cytokines and lineage-specific factors. IL-17A dramatically enhances erythropoiesis, increasing the production of ProE precursors above levels generated by Epo alone. HSC, hematopoietic stem cell; MPP, multipotent progenitor; EBMP, erythroid/basophil-mast cell/megakaryocytic progenitor; BFU-E, erythroid burst-forming unit; CFU-E, erythroid colony-forming unit; ProE, pro-erythroblast; BasoE, basophilic erythroblast; PolyE, poly erythroblast; OrthoE, ortho-chromatic erythroblast; RBC, red blood cell. Created in BioRender. Wilson, N. (2025) https://BioRender.com/naf26rb.

These results will be important in the search for new therapeutic approaches for the treatment of ineffective erythropoiesis, and highlight the need for combinatorial approaches in the search for “new” regulators.

In terms of potential limitations of the study, the focus is purely on one additional surface receptor and its ligand, whilst a previous study characterizes the effect of Mst1r, Ryk, and IL17RA and their respective ligands [6]. A more comprehensive analysis of the mechanistic underpinnings of the in vitro and in vivo results would expand on the applicability of the broader model, as exactly how IL17RA activation amplifies EPOR signaling remains unanswered. Therapeutic approaches for the treatment of anemia will not be trivial, but cross-species analysis of the experimental approaches used by this group would be of benefit and may also highlight additional ways of amplifying already existing gene programmes, rather than the need to identify novel pathways regulating these lineage decisions. Additional investigation would also be of use in a human: mouse transplant settings; for example, would the introduction of IL-17A improve erythroid output in this “stress” environment, or could the administration of an inflammatory cytokine have undesirable effects. Finally, could these approaches of amplifying already existing settings also be used in the “manufacture” of red blood cells on demand, where the output and cost of current protocols are making commercial in vitro production of blood products not (yet) viable.
